# AUTOMATIC CLASSIFICATION OF ADRD CAREGIVERS’ ONLINE INFORMATION WANTS: A MACHINE LEARNING APPROACH

**DOI:** 10.1093/geroni/igac059.1869

**Published:** 2022-12-20

**Authors:** Yuelyu Ji, Ning Zou, Bo Xie, Daqing He, Zhendong Wang

**Affiliations:** University of Pittsburgh, Pittsburgh, Pennsylvania, United States; University of Pittsburgh, Pittsburgh, Pennsylvania, United States; The University of Texas at Austin, Austin, Texas, United States; University of Pittsburgh, Pittsburgh, Pennsylvania, United States; University of Pittsburgh, Pittsburgh, Pennsylvania, United States

## Abstract

Social media platforms are used by caregivers for persons with Alzheimer’s Disease and its Related Dementias (ADRD) to obtain care information. Understanding what types of information caregivers want is critical to developing interventions to provide tailored information. Existing research on caregivers’ information wants has relied primarily on manual data analysis, unable to handle the vast amount of data available on social media. In our prior work, we adapted the validated Health Information Wants (HIW) framework to the ADRD caregiving context, forming the HIW-ADRD framework that includes 7 types of information commonly wanted by caregivers. Our longer-term goal is to develop machine algorithms that use the HIW-ADRD framework to automatically classify caregivers’ information wants from vast social media posts. Towards this end, we first scrapped posts from ADRD-related subgroups on Reddit, a popular social media platform. We then used few-shot learning, a machine learning method, to extract HIW from the posts. Using questions with question marks as a primary indicator for HIW, we filtered out those sentences and their corresponding background information. Next, we combined the questions and their background information as summaries of the posts. Finally, we sent the summaries to a classification model that classified these summaries based on HIW categories. We used 200 annotated posts to train the model, then tested it on 16779 posts. The evaluation results showed that our model achieved 62.35% in accuracy. These findings provide preliminary evidence for both the deep learning process and the algorithms. This study has implications for future research.

